# Data on the relationship between acetone, ethylene glycol, isopropanol, methanol, and propylene glycol serum/plasma concentrations and osmolal gaps in patients at an academic medical center

**DOI:** 10.1016/j.dib.2020.105189

**Published:** 2020-01-27

**Authors:** Heather R. Greene, Matthew D. Krasowski

**Affiliations:** a Carver College of Medicine, University of Iowa, IA, USA; b Department of Pathology, University of Iowa Hospitals and Clinics, 200 Hawkins Drive, Iowa City, IA, 52242, USA

**Keywords:** Acetone, Ethylene glycol, Isopropanol, Methanol, Osmolality, Propylene glycol, Toxicology

## Abstract

Ingestion of toxic alcohols other than ethanol (ethylene glycol, methanol, isopropanol, and propylene glycol) can cause life-threatening complications including altered level of consciousness, respiratory depression, and organ damage from metabolites. Many hospitals lack the ability to specifically analyze these compounds using gas chromatography, gas chromatography/mass spectrometry, or by enzymatic assays for ethylene glycol. Consequently, the presence of these compounds in blood is often ascertained indirectly by laboratory testing for acid-base status, osmolal gap, and anion gap. In the related research article, we analyzed 260 samples originating from 158 unique patients that had osmolal gap and specific testing for toxic alcohols performed on serum/plasma at an academic medical center central clinical laboratory. The data in this article provide the patient demographic, osmolal gap (and associated laboratory tests needed for this calculation), ethanol concentration by enzymatic assay, specific testing for toxic alcohols (ethylene glycol, isopropanol, methanol, propylene glycol) and acetone, anion gap, clinical history, antidotal treatment, and estimated timing of ingestion. The analyzed data is provided in the supplementary tables included in this article. Bias plots of osmolal gap estimations are included in a figure. The dataset reported is related to the research article entitled “Correlation of Osmolal Gap with Measured Concentrations of Acetone, Ethylene Glycol, Isopropanol, Methanol, and Propylene Glycol in Patients at an Academic Medical Center” [1].

## Highlights

• The osmolal gap estimated from measured concentrations of toxic alcohols tends to underestimate the traditional osmolal gap.
• Elevated anion gaps are common in patients ingesting ethylene glycol, isopropanol, and methanol.
• Activated charcoal administration was a common cause of propylene glycol detected in serum/plasma.

**Table:** Specifications Table

Subject	Medicine and Dentistry
Specific subject area	Pathology and Medical Technology
Type of data	Supplementary tables Figure
How data were acquired	Retrospective chart and data review from laboratory analysis performed at an academic medical center central clinical laboratory
Data format	Raw and Analyzed
Parameters for data were collection	Retrospective data was obtained from the electronic medical record (Epic, Inc.) covering the time period from November 1, 1996 through May 31, 2019. Detailed chart review was performed for all records. The project had approval from the University of Iowa Institutional Review Board.
Description of data collection	There were a total of 260 measurements in 158 unique patients. Laboratory testing includes analysis of serum/plasma on Roche Diagnostics clinical chemistry analyzers (including enzymatic assays for ethanol and ethylene glycol) and also gas chromatography (GC) for acetone, ethanol, ethylene glycol, isopropanol, methanol, and propylene glycol. Data include patient location at time of testing, age in years, birth sex, clinical history related to ingestions, serum/plasma laboratory values and associated calculations (sodium, glucose, blood urea nitrogen, ethanol by enzymatic assay, measured osmolality, osmolal gap without correction for ethanol, osmolal gap with correction for ethanol, ethanol concentration by GC, methanol concentration by GC, isopropanol concentration by GC, ethylene glycol concentration by either GC or enzymatic assay, propylene glycol concentration by GC, anion gap), use of activated charcoal, and antidotal therapy for toxic alcohols (ethanol, fomepizole, hemodialysis).
Data source location	Iowa City, Iowa, United States of America
Data accessibility	Raw data are available in this article as a figure and 5 Supplementary files.
Related research article	Author's name Heather R. Greene, Matthew D. Krasowski, Title **Correlation of Osmolal Gap with Measured Concentrations of Acetone, Ethylene Glycol, Isopropanol, Methanol, and Propylene Glycol in Patients at an Academic Medical Center** Journal Toxicol Rep https://doi.org/10.1016/j.toxrep.2019.12.005.

**Table:** 

**Value of the Data** • The data provided are of value as toxic alcohols continue to be a public health risk, and there are only limited published data sets that include detailed clinical history and raw data. • Clinicians, other researchers, or personnel in clinical laboratories might find this data useful as a reference for comparison. • Our data set would serve as a starting point for researchers interested in future investigations investigating the clinical utility and limitations of osmolal and anion gaps for diagnosis and management of toxic alcohol poisonings. • The data are of value as there is very limited published data involving the relationship of osmolal gaps to serum/plasma concentrations of isopropanol and acetone in patients. • The data provide information for 260 measurements in 158 unique patients.

## 1 Data

**Fig. 1:**
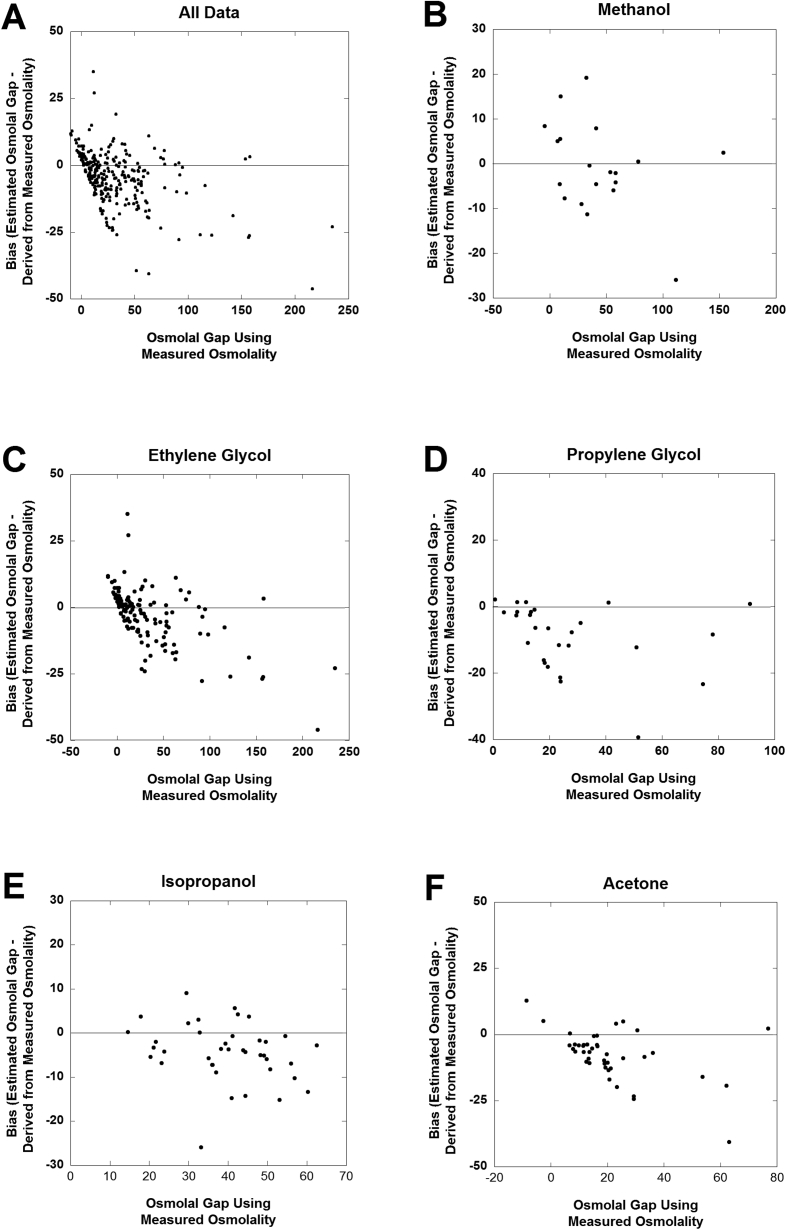
Bias plots of osmolal gap calculated by traditional route using measured osmolality vs. osmolal gap estimated from measured values of toxic alcohols and glycols using conversion factors. **A**. Combines all 260 measurements, while **B–F**. Divide the data by primary ingestion (B, methanol; C, ethylene glycol; D, propylene glycol; E, isopropanol; F, acetone).

In this retrospective analysis study, we compiled detailed data on 260 samples originating from 158 unique patients that had osmolal gap and specific testing for toxic alcohols performed on serum/plasma at an academic medical center central clinical laboratory [1]. Ingestion of toxic alcohols and glycols continues to be a significant clinical problem [[2], [3], [4], [5], [6], [7]]. Many clinical laboratories do not perform specific analysis for alcohols or glycols other than ethanol. Thus, indirect measures such as anion and osmolal gap are commonly used, even though these have some limitations [[8], [9], [10]]. The raw data are included in Supplementary file 1 (acetone), Supplementary file 2 (ethylene glycol), Supplementary file 3 (isopropanol), Supplementary file 4 (methanol), Supplementary file 5 (propylene glycol). Fig. 1 shows bias plots of osmolal gap calculated by traditional route using measured osmolality minus osmolal gap estimated from measured values of toxic alcohols and glycols using conversion factors.

• Supplementary file 1: Data for 42 measurements on 36 unique patients (11 female, 25 male) for which acetone was the primary compound detected by gas chromatography (GC). All laboratory data involve analysis on serum/plasma. Specific data fields include: location/unit at time of testing (emergency department or inpatient), age in years, birth sex, sodium concentration (mEq/L), glucose concentration (mg/dL), blood urea nitrogen (mg/dL), ethanol concentration by enzymatic assay (mg/dL), measured osmolality (mOsm/kg), osmolal gap without correction for ethanol, osmolal gap with correction for ethanol, estimated osmolal contribution of alcohols and glycols, methanol concentration (mg/dL) by GC, isopropanol concentration (mg/dL) by GC, ethanol concentration (mg/dL) by GC, acetone concentration (mg/dL) by GC, ethylene glycol concentration (mg/dL) by either GC or enzymatic assay, propylene glycol concentration (mg/dL) by GC, anion gap (if available), anion gap greater than 16 (yes/no/unknown), whether the laboratory studies were the initial measurements during the hospital admission or emergency department visit, clinical history related to the ingestion or other explanation for toxic alcohols, whether the patient expired during the admission, whether intravenous ethanol was used as antidote, whether fomepizole was used as antidote, whether hemodialysis was used, whether activated charcoal was administered, and estimated timing of laboratory studies relative to ingestion (if known).
• Supplementary file 2: Data for 133 measurements on 53 unique patients (20 female, 33 male) for which ethylene glycol was the main toxic alcohol ingestion. Specific data elements are the same as for Supplementary file 1.
• Supplementary file 3: Data for 39 measurements on 30 unique patients (18 female, 12 male) for which isopropanol was the main toxic alcohol ingestion. Specific data elements are the same as for Supplementary files 1 and 2.
• Supplementary file 4: Data for 19 measurements on 12 unique patients (3 female, 9 male) for which methanol was the main toxic alcohol ingestion. Specific data elements are the same as for Supplementary files 1-3.
• Supplementary file 5: Data for 27 measurements on 27 unique patients (15 female, 12 male) for which propylene glycol was the primary compound detected by GC. Specific data elements are the same as for Supplementary files 1-4.

## 2 Experimental design, materials, and methods

Serum/plasma blood urea nitrogen, electrolytes, ethanol by enzymatic assay, and ethylene glycol by enzymatic assay were determined on Roche Diagnostics chemistry analyzers. Serum/plasma osmolality was determined on an osmometer by freezing point depression. Specific analysis of acetone, ethanol, ethylene glycol, isopropanol, methanol, and propylene glycol was achieved using GC, with a lower limit of quantitation of 10 mg/dL. Enzymatic assay analysis for ethylene glycol was available in the hospital clinical laboratory starting in October 2010 [11,12]. Epic Reporting Workbench (RWB), a reporting tool within the electronic medical record [8], was used to capture all cases where osmolal gap had been determined and specific analysis for toxic alcohols and/or glycols had been performed. The conversion factors for estimating toxic alcohol and acetone concentrations in mg/dL by multiplying OG by the conversion factor are: acetone, 5.8; ethylene glycol, 6.2; isopropanol, 6.0; methanol, 3.2; and propylene glycol, 7.6 [2,3,7,13]. The authors performed detailed chart review for clinical history, ingestion details, and treatment.
